# Integrated Transcriptomic and Metabolic Analyses Reveal Key Defense Pathways Against *Fusarium* Infection in Maize Kernels

**DOI:** 10.3390/plants15081148

**Published:** 2026-04-09

**Authors:** Yuying Jia, Xin Qi, Xinfang Liu, Jun Ma, Mo Zhang, Chengtao Sun, Zhiyan Cao, Chunsheng Xue, Yanbo Wang

**Affiliations:** 1 State Key Laboratory of Maize Bio-Breeding, Liaoning Academy of Agricultural Sciences, Shenyang 110161, China; jiayuyinggood@163.com (Y.J.);; 2 College of Plant Protection, Shenyang Agricultural University, Shenyang 110866, China; 3 College of Plant Protection, Hebei Agricultural University, Baoding 071001, China

**Keywords:** maize, *Fusarium* ear rot, phenylpropanoid pathway, lignin biosynthesis

## Abstract

*Fusarium* ear rot (FER), caused by *F. verticillioides*, is a devastating disease in maize, leading to substantial yield losses and mycotoxin contamination. Therefore, revealing the molecular mechanisms underlying FER resistance is essential for crop breeding. Here, we performed integrated transcriptomic and metabolomic analyses on two maize inbred lines with contrasting FER resistance: the resistant line ZL30-12 (ZL30) and the susceptible line 92C0468U (92C). Following *F. verticillioides* inoculation, ZL30 exhibited sustained inhibition of fungal colonization and fumonisin accumulation, whereas 92C showed progressive disease development and elevated fumonisin levels. Both transcriptomic and metabolomic analyses converged on the phenylpropanoid pathway, with DEGs enriched in phenylpropanoid metabolism and DAMs enriched in phenylpropanoid biosynthesis, highlighting its central role in resistance. Further integrative analysis revealed that the lignin biosynthetic process, a key branch of phenylpropanoid metabolism, plays an important role in resistance. Several key DEGs (*ZmPAL*, *ZmHCT*, *peroxidases*, and *ZmCOMT*) and DAMs (sinapic acid, sinapaldehyde, coniferin, cinnamic acid, and caffeic acid) were differentially regulated between the two lines. Correlation analysis revealed a significant correlation between *ZmCOMT* expression and sinapic acid accumulation. RT-qPCR validation confirmed the expression patterns of key lignin-associated genes. The elevated activation of lignin biosynthesis in ZL30, via time-dependent induction of key genes (*ZmPAL*, *ZmHCT*, and *peroxidases*), suggests an increase in lignin accumulation, which likely reinforces cell wall integrity and restricts fungal invasion, thereby contributing to FER resistance. Collectively, these findings provide insights into the molecular mechanisms of FER resistance and identify key lignin-associated genes as promising targets for maize breeding.

## 1. Introduction

Maize (*Zea mays* L.) is a major global crop for food, feed, and industrial applications, playing a critical role in agricultural production and economic development worldwide. *Fusarium* ear rot (FER), one of the most destructive diseases of maize, causes an estimated 10–20% yield loss annually and up to 50% or more in severely infected regions [1]. This disease is mainly caused by several *Fusarium* spp., among which *F. verticillioides* is the predominant pathogen due to its high natural prevalence [2]. *F. verticillioides* produces various mycotoxins, including beauvericin, fusarin C, fumonisins, and moniliformin, with fumonisins being the primary toxic metabolites [3]. Among fumonisins, fumonisin B1 (FB1) is more abundant than fumonisins B2 (FB2) and B3 (FB3) [4]. These mycotoxins not only pose severe threats to livestock health but also increase the risk of fatal cancers in humans [5,6]. Kernels infected by *F. verticillioides* are characterized by random white to light pink fungal growth and white streaks radiating across the kernel surface [7]. FER is prevalent in warm and dry regions, with an optimum temperature for pathogen infection of approximately 30 °C [8,9]. In China, FER was first reported in Henan Province and has now spread to all major maize-growing areas [10]. Therefore, developing effective and sustainable strategies to control FER is urgently needed.

FER resistance is a typical complex quantitative trait, primarily controlled by multiple minor-effect quantitative trait loci (QTLs) and strongly influenced by environmental factors. These QTLs exhibit additive, dominant, and additive-dominant epistatic effects, with epistatic effects playing a pivotal role in regulating resistance [11,12,13]. Over the past decades, extensive QTL mapping studies have been conducted using diverse maize populations, and resistance QTLs have been identified across all 10 maize chromosomes [13]. Transcriptomic studies have further uncovered key functional genes underlying FER resistance. For instance, *ZmMPK3* was identified as a core resistance gene [14], and loss of function of *ZmWRKY125* was shown to enhance resistance by activating jasmonic acid and abscisic acid signaling pathways [15]. Additionally, *ZmSIZ1a* and *ZmSIZ1b* were found to mediate resistance by modulating early defense and MAPK signaling pathways [16]. However, the molecular mechanisms underlying FER resistance remain largely unclear, and more efficient strategies are required to identify key defense pathways and resistance-related genes.

The phenylpropanoid pathway is a core secondary metabolic pathway in higher plants and serves as the exclusive upstream regulatory pathway for lignin biosynthesis, producing various phenolic polymers and lignin as major products. These metabolites and lignin biosynthetic intermediates contribute to disease resistance mainly through two mechanisms: strengthening the cell wall via lignin deposition and producing antimicrobial phenolic compounds [17,18,19]. Key genes in the phenylpropanoid pathway, such as those encoding phenylalanine ammonia lyase (PAL), hydroxycinnamoyl transferase (HCT), and caffeic acid O-methyltransferase (COMT), are essential for the biosynthesis of defense-related metabolites and act as core regulators of plant defense responses against pathogens [20]. Although the phenylpropanoid–lignin pathway has been implicated in resistance to various fungal diseases in plants [21], its molecular mechanism and regulatory role in FER resistance remain to be fully elucidated.

Transcriptome analysis serves as a powerful tool for identifying candidate resistance genes by analyzing differential expression across genotypes and time points, and it has been widely used to dissect disease resistance mechanisms [22,23,24]. As a critical complement to transcriptomic studies, metabolomics provides an indispensable approach for identifying resistance-related candidate metabolites [25,26]. With the rapid development of omics technologies, integrated transcriptomic and metabolic analyses have emerged as powerful strategies to dissect the complex regulatory networks and identify key genes and metabolites involved in plant defense responses [27]. These integrative approaches have been extensively applied in plant–pathogen interaction studies, facilitating the discovery of key resistance-related genes and critical metabolic pathways underlying plant immunity.

In this study, we performed an integrated metabolomic and transcriptomic analysis of kernels from ZL30-12 (ZL30) and 92C0468U (92C) at different time points after *F. verticillioides* inoculation. The main objectives were to (1) characterize the specialized metabolite composition in the two maize inbred lines during *F. verticillioides* infection and (2) identify key metabolic pathways and regulatory genes involved in maize defense responses to FER.

## 2. Results

### 2.1. Resistance Evaluation and Fumonisin Analysis of ZL30 and 92C

**Figure 1:**
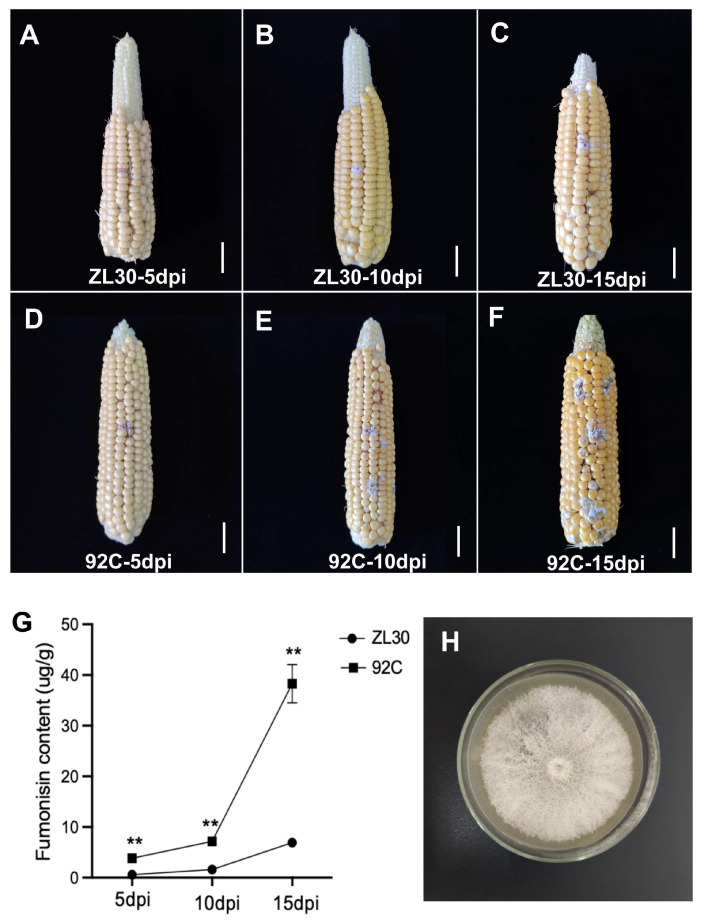
Phenotypic evaluation of ZL30 and 92C infected with *F. verticillioides*. Phenotypes of maize ears in ZL30 at 5 dpi (**A**), 10 dpi (**B**), and 15 dpi (**C**) and 92C at 5 dpi (**D**), 10 dpi (**E**), and 15 dpi (**F**); scale bar = 2 cm. (**G**) Fumonisin content (FB1, FB2, and FB3) in *F. verticillioides* inoculated kernels of ZL30 and 92C at 5, 10, and 15 dpi. ** indicates significant differences (*p* < 0.01). (**H**) Colony morphology of *F. verticillioides* on PDA medium.

Following inoculation with *F. verticillioides*, ZL30 exhibited minimal disease symptoms across three time points, with kernels remaining plump and free of visible mold. At 15 days post-inoculation (dpi), the average diseased area percentage was markedly lower in ZL30 (2%) compared to 92C (21.4%) (Figure 1A–F and Figure S1A). The *F. verticillioides* strain used in this study exhibited typical morphological characteristics on potato dextrose agar (PDA) medium, with white mycelia (Figure 1H). To clarify the resistance phenotype of maize inbred lines to *F. verticillioides*, the total content of fumonisins (FB1 + FB2 + FB3) in the ears of the two lines was determined at different time points after inoculation. The results showed that the total fumonisin content in 92C increased significantly with the prolonged inoculation time and reached its peak at 15 dpi, while that in ZL30 remained at a low level throughout the whole period and was significantly lower than that in 92C at all time points (5, 10, and 15 dpi) (*p* < 0.01) (Figure 1G). To investigate the differential accumulation of fumonisins, we analyzed the temporal dynamics of the three major monomers (FB1, FB2, and FB3). The results showed that fumonisin B1 (FB1), the most toxic monomer, was the predominant form in 92C, with its content rising sharply from 5 to 15 dpi and peaking at 28.61 µg/g. Importantly, the levels of all three fumonisin monomers (FB1, FB2, and FB3) were consistently and significantly higher in 92C than in ZL30 at every time point (*p* < 0.01) (Figure S1B–D). The accumulation pattern of FB1 closely mirrored that of total fumonisins, confirming that FB1 is the primary contributor to overall toxin accumulation. These results collectively demonstrate that the resistant line ZL30 effectively inhibits *F. verticillioides* infection and suppresses the accumulation of fumonisins, particularly the predominant and most toxic monomer FB1, thus exhibiting a resistant phenotype against *Fusarium* ear rot (FER).

### 2.2. Transcriptome Profiling of ZL30 and 92C in Response to F. verticillioides

**Figure 2:**
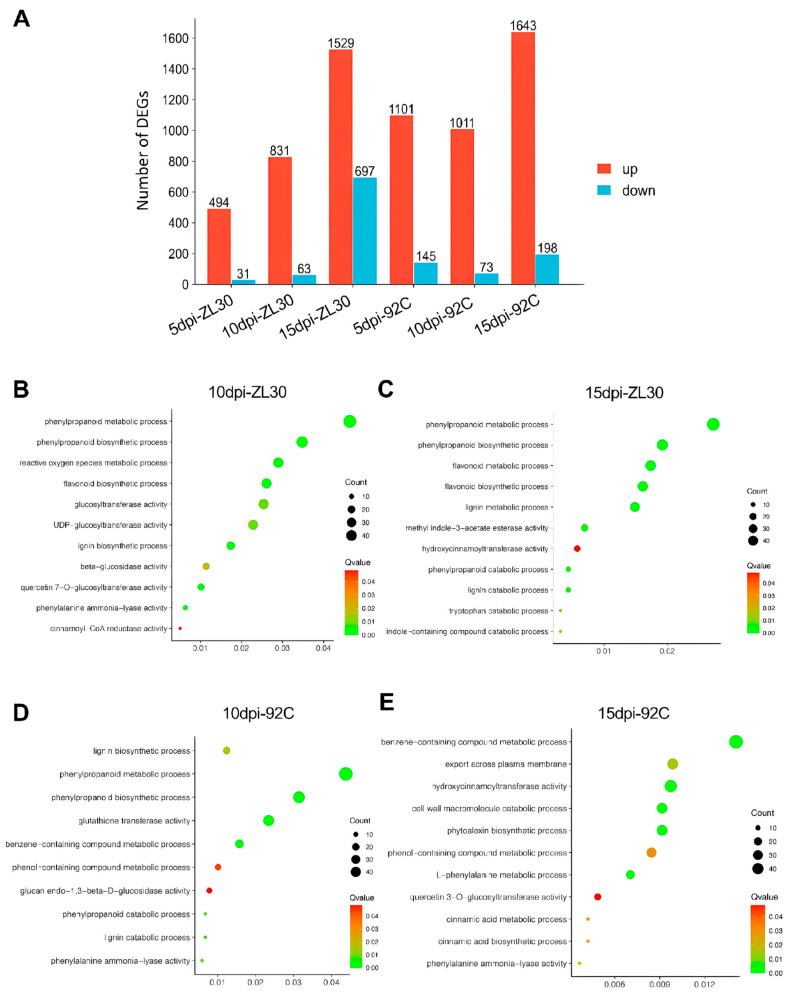
Transcriptomic profiling of ZL30 and 92C at different post-inoculation time points. (**A**) Number of differentially expressed genes (DEGs) in ZL30 and 92C at 5, 10, and 15 dpi; (**B**–**E**) GO enrichment analysis of DEGs in ZL30 in response to *F. verticillioides* inoculation at 10 dpi (**B**) and 15 dpi (**C**); GO enrichment analysis of DEGs in 92C in response to *F. verticillioides* inoculation at 10 dpi (**D**) and 15 dpi (**E**).

To investigate the molecular responses of maize to *F. verticillioides* infection, we performed comparative transcriptome analysis of two maize inbred lines at different time points post-inoculation (5, 10, and 15 dpi). A substantial number of differentially expressed genes (DEGs) were identified in ZL30 and 92C in both water-inoculated (CK) and *F. verticillioides*-inoculated samples across the three time points. We obtained the highest number of DEGs (2222) between water-inoculated ZL30 (CK) and pathogen-inoculated ZL30 (CK vs. ZL30) at 15 dpi. Among these DEGs, 1529 were upregulated, and 697 were downregulated, whereas only 525 DEGs (494 upregulated and 31 downregulated) were detected in ZL30 at 5 dpi. Moreover, 894 DEGs (831 upregulated and 63 downregulated) were identified at 10 dpi. Similarly, the susceptible line 92C (CK vs. 92C) exhibited a progressive increase in DEGs, with the highest number (1643 upregulated and 198 downregulated) at 15 dpi, followed 1246 DEGs (1101 upregulated and 145 downregulated) at 5 dpi and 1048 DEGs (1011 upregulated and 73 downregulated) at 10 dpi (Figure 2A and Tables S1–S6). These results indicate that transcriptional responses in both lines intensify progressively during infection and peak at 15 dpi.

To explore the underlying biological processes modulated in response to *F. verticillioides* infection, Gene Ontology (GO) enrichment analysis was performed on the DEGs identified at each time point in both lines. In ZL30, DEGs at 5 dpi were significantly enriched in terms related to “monooxygenase activity”, “phenylpropanoid metabolic process”, and “reactive oxygen species metabolic process”, suggesting an immediate activation of defense-related pathways (Figure S2A). By 10 dpi, enriched terms further highlighted “phenylpropanoid biosynthetic process”, “flavonoid biosynthetic process”, “glucosyltransferase activity”, and “lignin biosynthetic process” (Figure 2B), indicating a sustained and coordinated activation of specialized metabolic pathways. At 15 dpi, phenylpropanoid and lignin-related metabolic pathways (“phenylpropanoid metabolic process” and “phenylpropanoid biosynthesis process”) remained the most significantly enriched terms, with the highest enrichment scores among all biological processes (Figure 2C). In contrast, the susceptible line 92C exhibited a delayed and less coordinated enrichment pattern. Although terms such as “phenylpropanoid metabolic process” and “phenylpropanoid biosynthetic process” were enriched at 5 dpi (Figure S2B), key pathways related to “lignin biosynthetic process” were weakly enriched at 10 dpi compared with ZL30 (Figure 2D). By 15 dpi, the enrichment profile showed only limited activation of defense-related pathways in 92C, and “benzene-containing compound metabolic process” was detected, whereas core phenylpropanoid biosynthetic terms (“phenylalanine ammonia-lyase activity” and “cinnamic acid biosynthetic process”) were only weakly enriched (Figure 2E). These results suggest that the phenylpropanoid metabolic pathway likely plays a key role in mediating maize resistance to FER, with the resistant line showing immediate and sustained activation, whereas the susceptible line exhibits a delayed and less coordinated activation pattern.

### 2.3. Comparative Metabolic Profiling of ZL30 and 92C upon F. verticillioides Inoculation

**Figure 3:**
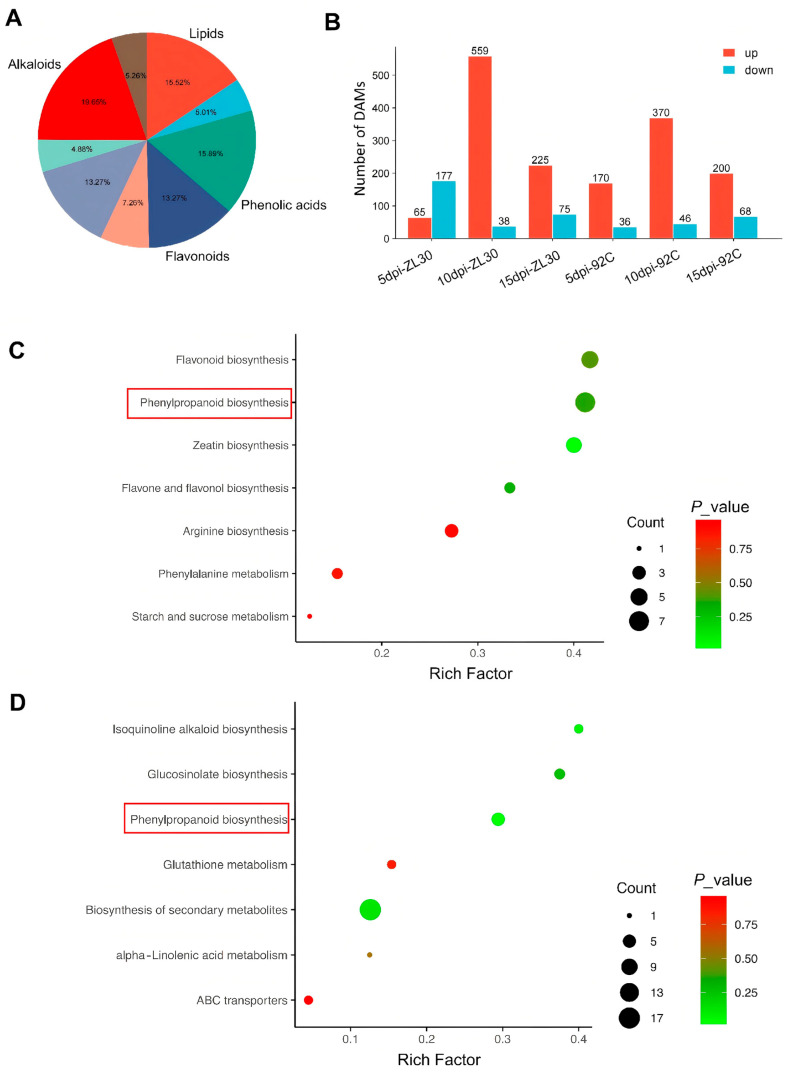
Metabolic profiling of ZL30 and 92C at different post-inoculation time points. (**A**) Category and number of the metabolites identified in the two maize inbred lines; (**B**) number of differentially accumulated metabolites in ZL30 and 92C at 5, 10, and 15 dpi; (**C**,**D**) KEGG pathway enrichment of DAMs in ZL30 (**C**) and 92C (**D**) inbred lines. The red boxes mark the phenylpropanoid biosynthesis pathway, commonly enriched pathway in both ZL30 and 92C.

To assess the reproducibility of the metabolomic data, principal component analysis (PCA) was performed on all samples. Biological replicates clustered tightly within each treatment group (Figure S3), demonstrating the repeatability and reliability of the metabolomic data. To investigate the dynamic changes in metabolite contents between ZL30 and 92C after *F. verticillioides* infection, kernels of the two lines were separately subjected to *F. verticillioides* inoculation and mock inoculation with sterile water. Kernels were collected at 5, 10, and 15 dpi for metabolomic analysis. The metabolites identified in the two lines were classified into several major categories, with alkaloids (19.65%), phenolic acids (15.89%), lipids (15.52%), and flavonoids (13.27%) as the predominant classes, collectively accounting for the majority of the detected metabolites (Figure 3A). To identify metabolites associated with FER resistance, we further compared the metabolic profiles between ZL30 and 92C at each post-inoculation time point. The highest number of differentially accumulated metabolites in ZL30 was observed at 10 dpi, with 559 upregulated and 38 downregulated (Table S7). Meanwhile, 65 metabolites were upregulated and 177 downregulated at 5 dpi (Table S8), while 225 were upregulated and only 75 downregulated at 15 dpi (Table S9). In 92C, the peak number of differentially accumulated metabolites (DAMs) was also detected at 10 dpi, comprising 370 upregulated and 46 downregulated (Table S10). At 5 dpi, 170 metabolites were upregulated and 36 downregulated (Table S11), whereas 200 were upregulated and 68 downregulated at 15 dpi (Figure 3B and Table S12). Together, these temporal profiles suggest that both lines exhibit their strongest metabolic responses at 10 dpi. The resistant line ZL30 sustains a more coordinated upregulation of metabolites during late post-inoculation stages, whereas the susceptible line 92C shows a progressive increase in downregulated metabolites, indicative of deteriorating metabolic homeostasis under prolonged pathogen stress.

To elucidate the biological pathways underlying the observed metabolic profiles, Kyoto Encyclopedia of Genes and Genomes (KEGG) pathway enrichment analysis was performed on the DAMs identified in ZL30 and 92C. DAMs were enriched in pathways related to “Flavonoid biosynthesis (ko00941)”, “Phenylpropanoid biosynthesis (ko00940)”, “Zeatin biosynthesis (ko00908)”, and “Flavone and flavonol biosynthesis (ko00944)” in ZL30 (Figure 3C). In 92C, DAMs were predominantly enriched in pathways including “Isoquinoline alkaloid biosynthesis (ko00950)”, “Glucosinolate biosynthesis (ko00966)”, “Phenylpropanoid biosynthesis (ko00940)”, and “Biosynthesis of secondary metabolites (ko01110)” (Figure 3D). Notably, “phenylpropanoid biosynthesis (ko00940)” was identified as a commonly enriched pathway in both lines, highlighting its potential role in the metabolic response to *F. verticillioides* infection. This common enrichment indicates that “phenylpropanoid metabolism” serves as a core defense-associated metabolic response in ZL30 and 92C against FER.

### 2.4. DAMs and DEGs Involved in the Phenylpropanoid Metabolic Pathway

**Figure 4:**
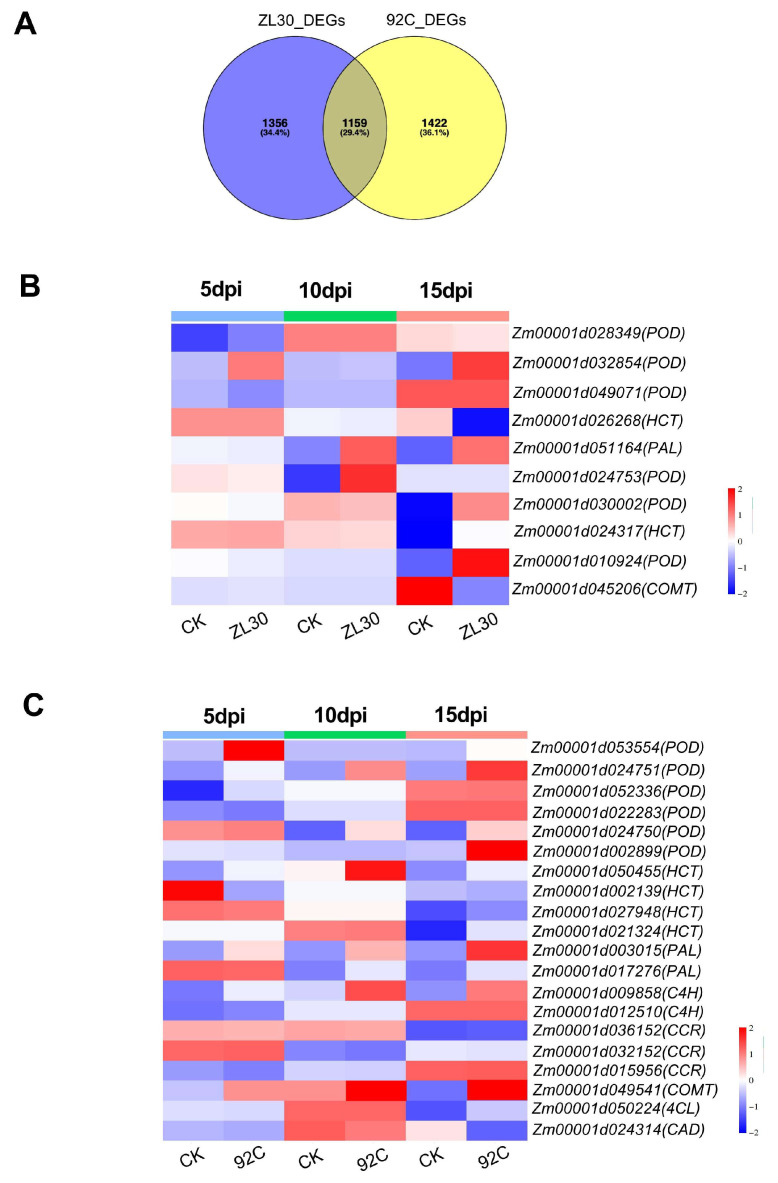
Specific differentially expressed genes (DEGs) unique to ZL30 and 92C. (**A**) Venn diagram of specific DEGs in ZL30 and 92C; (**B**,**C**) heatmap of specific DEGs in the phenylpropanoid metabolic pathway of ZL30 (**B**) and 92C (**C**); CK (water inoculated), ZL30 *(F. verticillioides* inoculated) and 92C (*F. verticillioides* inoculated). The color bars at the top of the heatmap indicate different time points after inoculation: blue for 5 dpi, green for 10 dpi, and pink for 15 dpi.

Since “phenylpropanoid metabolism” was enriched in both lines in the preceding analysis, we focused on characterizing the expression and accumulation patterns of DEGs and DAMs in this pathway to further dissect the molecular mechanisms underlying maize resistance to FER. To identify resistance-specific genes and metabolites associated with FER, we performed a Venn analysis of DEGs across comparison groups. The analysis revealed 1356 and 1422 unique DEGs in ZL30 and 92C, respectively (Figure 4A and Table S13), suggesting that these genes may function as core regulatory components specific to resistance or susceptibility. Key phenylpropanoid pathway genes exhibited distinct time-dependent expression profiles in ZL30. Peroxidase genes showed early induction (*Zm00001d028349*, upregulated at 5 dpi) or sustained induction (*Zm00001d032854*, upregulated at 5 and 15 dpi), while *Zm00001d049071* was downregulated at the early stage. *PAL* (*Zm00001d051164*) and *HCT* (*Zm00001d024317*) were significantly upregulated at the later stages, whereas *COMT* (*Zm00001d045206*) was downregulated at 15 dpi (Figure 4B). In 92C, several genes including peroxidase gene (*Zm00001d024751*), *PAL* (*Zm00001d003015*), *HCT* (*Zm00001d050455*), *C4H* (*Zm00001d009858*), and *COMT* (*Zm00001d049541*) were upregulated at 5 dpi and maintained elevated expression through 15 dpi. In contrast, genes such as *HCT* (*Zm00001d002139*) and a peroxidase gene (*Zm00001d022283*) were downregulated at 5 dpi, while other genes, including *HCT* (*Zm00001d027948*), a peroxidase gene (*Zm00001d02899*), and *4CL* (*Zm00001d050224*), were induced only at the later stages (10 or 15 dpi) (Figure 4C).

**Figure 5:**
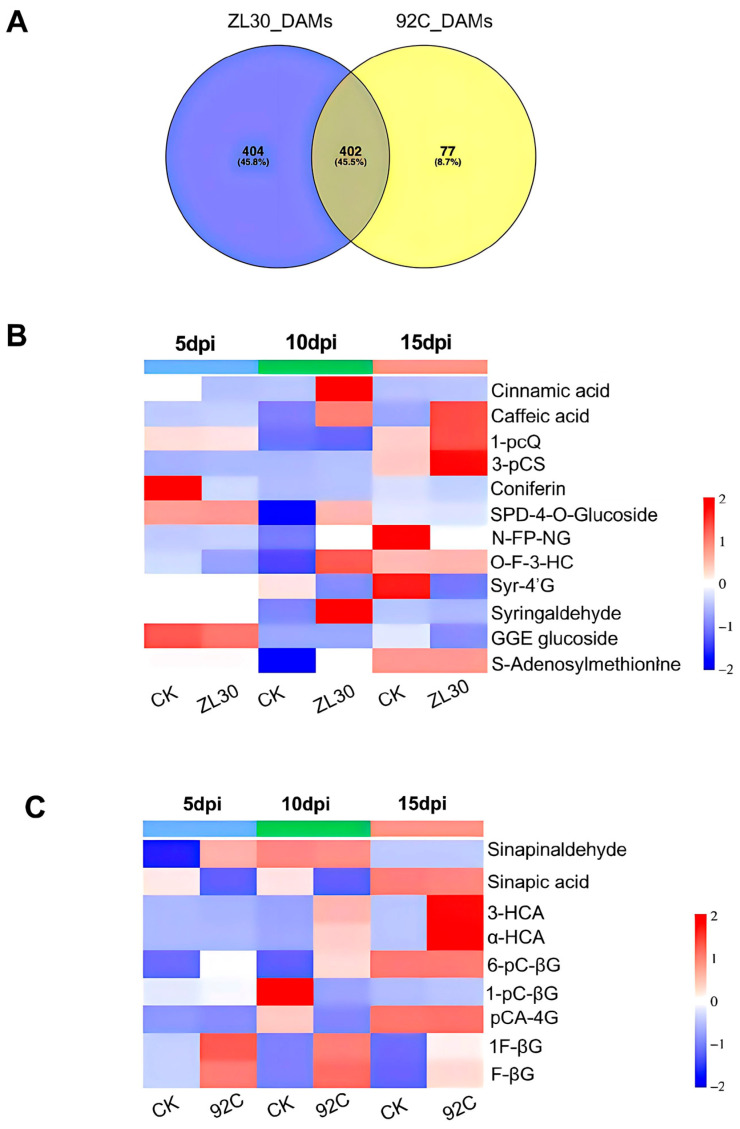
Specific differentially accumulated metabolites (DAMs) unique to ZL30 and 92C. (**A**) Venn diagram of specific DAMs in ZL30 and 92C; (**B**,**C**) heatmap of specific DAMs in the phenylpropanoid metabolic pathway of ZL30 (**B**) and 92C (**C**). CK (water inoculated), ZL30 (*F. verticillioides* inoculated) and 92C (*F. verticillioides* inoculated). The color bars at the top of the heatmap indicate different time points after inoculation: blue for 5 dpi, green for 10 dpi, and pink for 15 dpi.

To further characterize the phenylpropanoid pathway at the metabolic level, we analyzed the accumulation dynamics of DAMs in both lines at different time points. A Venn diagram identified 404 and 77 unique DAMs in the resistant and susceptible lines, respectively (Figure 5A and Table S14). In ZL30, two key upstream intermediates of lignin, cinnamic acid and caffeic acid, exhibited divergent accumulation patterns. Caffeic acid was upregulated at 10 and 15 dpi, while cinnamic acid was downregulated at 5 dpi and upregulated at 10 dpi (Figure 5B). Meanwhile, core precursors for lignin monomer synthesis showed distinct dynamics in 92C. Sinapinaldehyde (S-type lignin precursor) was upregulated at 5 dpi, whereas sinapic acid was downregulated at 5 and 10 dpi. 3-Hydroxycinnamic acid (3HCA) and α-hydroxycinnamic acid (αHCA) (H- and G-type lignin precursors) were upregulated at 10 and 15 dpi (Figure 5C). These results revealed significant differences in the accumulation of phenylpropanoid metabolites related to lignin biosynthesis between the two lines, suggesting that lignin biosynthesis is a key process in maize FER resistance.

To explore the upstream regulators driving differential activation of the phenylpropanoid pathway, we analyzed the expression patterns of phytohormone signaling and transcription factor genes. In resistant line ZL30, the jasmonic acid (JA) biosynthesis gene *LOX11* (*Zm00001d015852*) was significantly upregulated at 15 dpi (Table S3), along with sustained upregulation of a JA-responsive gene (*Zm00001d034068*) from 5 dpi onward (Tables S1–S3), indicating robust JA signaling activation. In contrast, susceptible line 92C exhibited a compromised JA response, with several *LOX* genes (*Zm00001d042540* and *Zm00001d040842*) downregulated at different time points (Tables S5 and S6). However, *MYB* transcription factors displayed opposite expression patterns between the two lines. In ZL30, several *MYB* genes (*Zm00001d005300* and *Zm00001d022259*) were downregulated (Tables S1–S3), whereas in 92C, the lignin biosynthesis regulator *MYB42* (*Zm00001d053220*) was upregulated at 15 dpi (Table S6). Thus, coordinated JA signaling and *MYB* repression enable timely phenylpropanoid activation in the resistant line, whereas the susceptible line presents dysregulated *MYB* expression and compromised defense.

### 2.5. The Role of Lignin Biosynthesis in Maize Resistance to Fusarium Ear Rot

**Figure 6:**
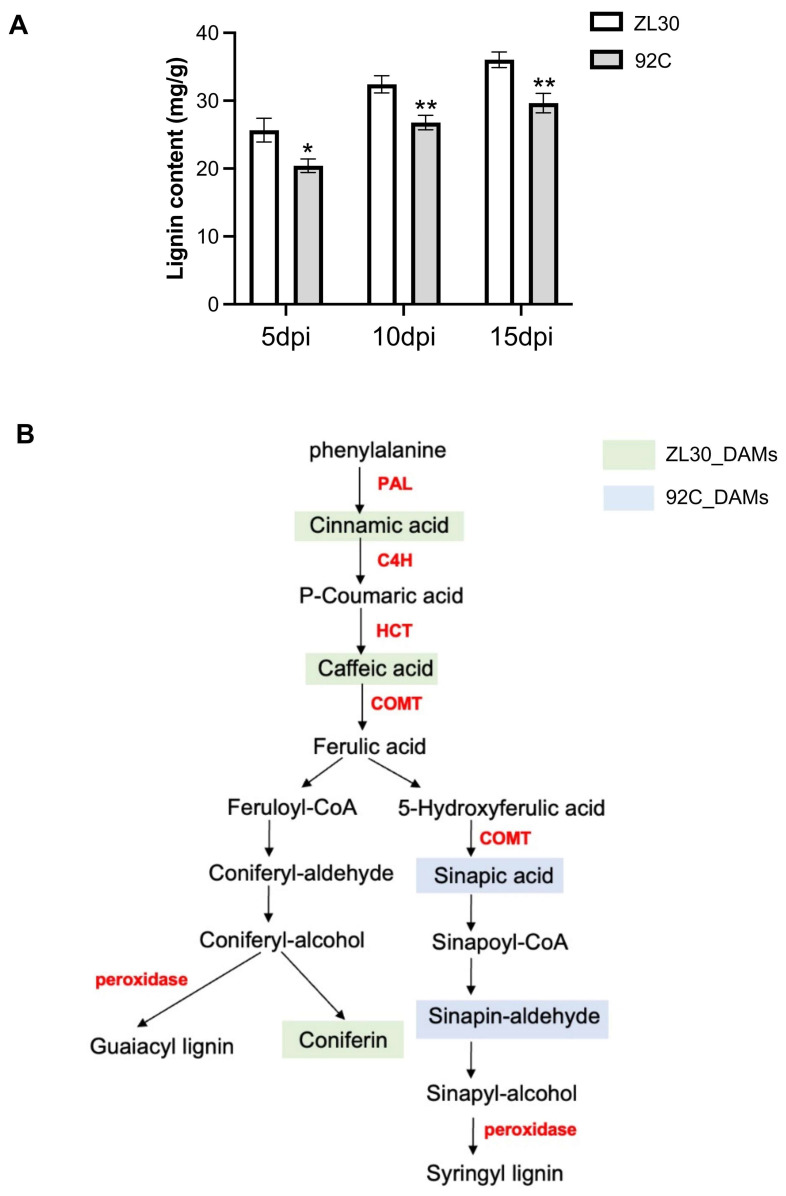
Lignin accumulation and differential regulation of the phenylpropanoid–lignin biosynthesis pathway in ZL30 and 92C following *F. verticillioides* inoculation. (**A**) Lignin content in ZL30 and 92C at 5, 10, and 15 days post-inoculation (dpi). Data are presented as the mean ± SD (*n* = 3). Asterisks indicate significant differences between ZL30 and 92C at each time point, as determined by Student’s *t*-test (*, *p* < 0.05; **, *p* < 0.01). (**B**) Schematic diagram of the phenylpropanoid–lignin biosynthesis pathway, showing differentially accumulated metabolites in ZL30 (light green) and 92C (light blue), with key genes (*PAL*, *C4H*, *HCT*, *COMT*, and peroxidase genes) involved in lignin biosynthesis highlighted in red.

Integrated transcriptomic and metabolic analysis suggests that lignin biosynthesis is critical for maize resistance to FER. To further validate the functional role of lignin biosynthesis in maize resistance to FER, the lignin content in ZL30 and 92C was determined at three time points (5, 10, and 15 dpi). The results showed that the lignin content in both lines increased over time and was consistently higher in ZL30 than in 92C at each time point (Figure 6A). Thus, a lignin biosynthesis pathway integrating key metabolites and genes was constructed based on the KEGG database, and these included five metabolites (cinnamic acid, caffeic acid, coniferin, sinapic acid, and sinapaldehyde) and several structural genes (*ZmPAL*, *ZmHCT*, *ZmCOMT*, and *peroxidases*) (Figure 6B). To achieve quantitative integration of genes and metabolites, we performed Pearson correlation analysis to directly link the expression of key lignin biosynthesis genes with the accumulation of their corresponding metabolites. The expression of *ZmCOMT* (*Zm00001d049541*) showed a negative correlation with sinapic acid (r = −0.92, *p* = 0.028) and a positive correlation with sinapaldehyde (r = 0.63, *p* = 0.021), consistent with its role in converting sinapic acid to sinapaldehyde in the monolignol biosynthesis pathway. Similarly, positive correlations were observed between *ZmPAL* (*Zm00001d051164*) and cinnamic acid (r = 0.75), and between *ZmHCT* (*Zm00001d024317*) and caffeic acid (r = 0.91) (Table S15), supporting the coordinated regulation of lignin biosynthesis. These correlations directly connect transcriptional regulation to metabolic flux in the lignin biosynthesis pathway. The resistant line ZL30 showed an immediate response with downregulation coniferin and cinnamic acid and induced peroxidase genes (*Zm00001d028349* and *Zm00001d032854*) at 5 dpi. The initial decrease in cinnamic acid was followed by its upregulation at 10 dpi, likely due to the sustained upregulation of *ZmPAL (Zm00001d051164*). Subsequently, the sustained accumulation of caffeic acid was driven by the upregulation of a specific *ZmHCT* gene (*Zm00001d024317*) at 15 dpi (Figure 4B and Figure 5B). In the susceptible line 92C, the upregulation of key early biosynthetic genes including *ZmPAL* (*Zm00001d003015*), *ZmHCT* (*Zm00001d050455*), and *ZmC4H* (*Zm00001d009858*) ensured the sufficient precursor supply of pathway precursors. Notably, the sustained upregulation of *ZmCOMT* (*Zm00001d049541*) was accompanied by a significant decrease in its substrate sinapic acid and a concomitant increase in the downstream intermediate sinapinaldehyde. Additionally, the specific induction of peroxidase genes (*Zm00001d053554*, *Zm00001d024751*, and *Zm00001d052336*) from 5 dpi indicated active lignin polymerization concurrent with these metabolic changes (Figure 4C and Figure 5C). These results demonstrate that divergent transcriptional and metabolic regulation of lignin biosynthesis accounts for the differential FER resistance between resistant and susceptible lines.

**Figure 7:**
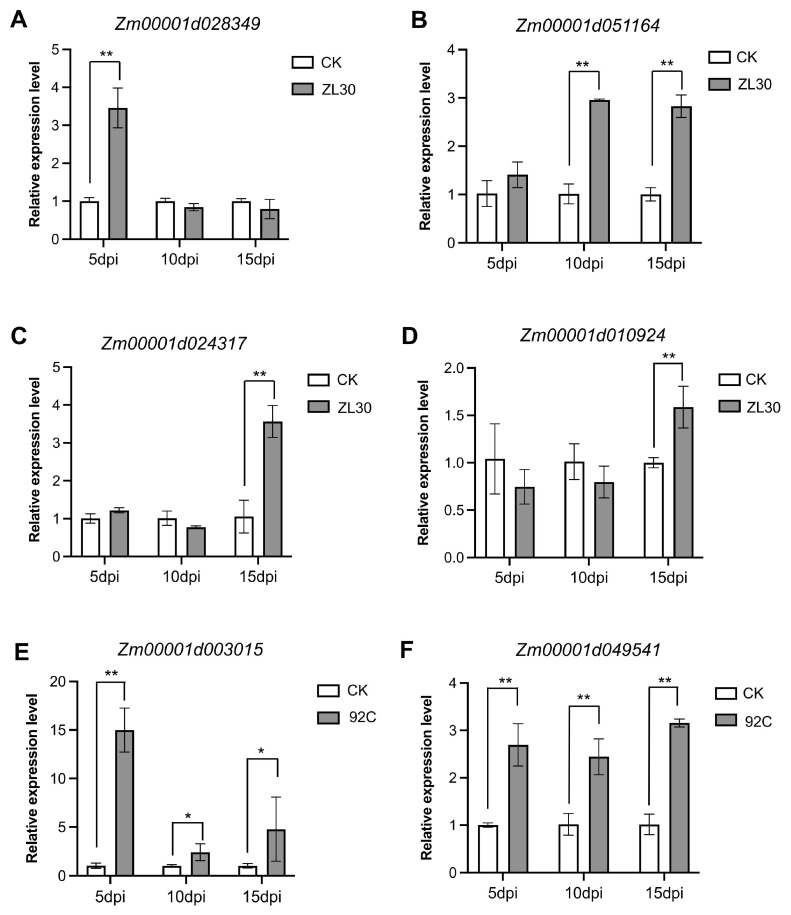
RT-qPCR validation of key differentially expressed genes (DEGs) involved in the phenylpropanoid–lignin biosynthesis pathway in ZL30 and 92C following *F. verticillioides* inoculation at 5, 10, and 15 dpi. Relative expression level of *Zm00001d028349* (**A**), *Zm00001d051164* (**B**), *Zm00001d024317* (**C**) and *Zm00001d010924* (**D**) in ZL30; relative expression level of *Zm00001d003015* (**E**) and *Zm00001d049541* (**F**) in 92C. Data are presented as the mean ± SD (*n* = 3). Asterisks indicate significant differences between CK (water-inoculated) and ZL30 (*F. verticillioides*-inoculated) or 92C (*F. verticillioides*-inoculated) (*, *p* < 0.05; **, *p* < 0.01).

To verify the reliability and reproducibility of the RNA-seq data, the expression levels of key genes involved in lignin biosynthesis were selected for qRT–PCR analysis. The results were consistent with the transcript profiles obtained from RNA-seq analysis. Genes significantly upregulated in the RNA-seq data were consistently upregulated in the RT-qPCR analysis, and downregulated genes showed a similar pattern. These findings further validate the reliability of the transcriptome sequencing data (Figure 7).

## 3. Discussion

Phenylpropanoids are important precursors of numerous structural polymers and defense compounds (such as lignin) in plants, serving as the core mediators of crosstalk between defense-related pathways [28]. The integrated transcriptomic and metabolomic analyses revealed that the phenylpropanoid pathway and lignin biosynthesis were specifically and continuously activated in the resistant line ZL30-12 (ZL30) upon *F. verticillioides* infection. Such early and sustained activation represents a typical characteristic of effective disease resistance, facilitating rapid cell wall reinforcement and stable production of defense metabolites. In contrast, the susceptible line 92C0468U (92C) exhibited delayed and uncoordinated induction of these defense processes, especially the lignin biosynthetic pathway, which may account for its inability to restrict pathogen invasion and mycotoxin accumulation. Consistently, metabolic KEGG enrichment analysis further verified the key role of the “phenylpropanoid biosynthesis” pathway in maize defense against *Fusarium* ear rot (FER). Collectively, these findings demonstrate that the “phenylpropanoid pathway” functions as an indispensable defense pathway in maize against *F. verticillioides*, and its timely, coordinated activation is essential for FER resistance.

The plant cell wall acts as the primary physical barrier against pathogen invasion [29], and lignin deposition represents a conserved defense strategy that reinforces cell wall integrity and blocks hyphal penetration [30]. In this study, the resistant line ZL30 exhibited significantly higher lignin accumulation, which was associated with reduced fungal colonization and suppressed fumonisin accumulation, especially the highly toxic fumonisn B1 (FB1). The increased lignin content in ZL30 likely contributes to physical reinforcement of the cell wall, thereby restricting fungal hyphal penetration and spread within kernel tissues. This physical barrier may also impede the translocation of fumonisins from infection sites to surrounding tissues, consistent with the significantly lower fumonisin levels detected in ZL30 compared to 92C across all time points. In contrast, the reduced lignin content in the susceptible line 92C likely compromises cell wall integrity, facilitating fungal colonization and subsequent mycotoxin accumulation and diffusion. These findings suggest that lignin-mediated cell wall reinforcement contributes to durable FER resistance by both blocking fungal hyphal penetration and limiting mycotoxin dissemination.

Lignin biosynthesis is tightly controlled by a series of key structural genes in the phenylpropanoid pathway, including *PAL*, *HCT*, *COMT*, and peroxidase genes [31,32,33]. Changes in the expression of these genes can redirect metabolic flux toward different lignin monomers, thereby directly affecting disease resistance [34,35]. In the resistant line ZL30, the timely induction of *PAL* (*Zm00001d051164*), *HCT* (*Zm00001d024317*), and peroxidase genes (*Zm00001d028349* and *Zm00001d032854*) was closely associated with coniferin downregulation at 5 dpi, cinnamic acid upregulation at 10 dpi, and the sustained accumulation of caffeic acid from 10 to 15 dpi. The downregulation of coniferin suggests enhanced flux toward G-type lignin biosynthesis. This sequential activation likely facilitates efficient lignin deposition and cell wall reinforcement. By contrast, in the susceptible line 92C, the early and sustained expression of several genes (*PAL*, *Zm00001d003015*; *HCT*, *Zm00001d050455*; *C4H*, *Zm00001d009858*; *COMT*, *Zm00001d049541*) was accompanied by a metabolic shift toward syringyl (S-type) lignin precursors and reduced sinapic acid levels, leading to an inefficient lignin polymerization process and potentially compromised cell wall structural integrity. Collectively, these results further confirmed that the lignin biosynthesis pathway is a core mechanism underlying maize defense against FER.

G-type lignin monomers and caffeoyl alcohol are biosynthesized by *HCT*, *C4H*, *CCR,* and *CAD*, whereas sinapyl alcohol and S-type lignin monomers are produced by a branched metabolic pathway mediated by *F5H*, *COMT*, and *CAD* [20]. The upregulation of *ZmHCT* (*Zm00001d024317*) in ZL30 was positively correlated with the accumulation of caffeic acid, indicating that *ZmHCT* plays a key role in directing metabolic flux toward lignin synthesis. PAL catalyzes the initial step of the general phenylpropanoid pathway by converting phenylalanine to cinnamic acid, and *PAL* genes have been consistently shown to contribute to broad-spectrum disease resistance through cell wall-mediated immunity [36]. The upregulation of *ZmPAL* (*Zm00001d051164*) may induce cinnamic acid accumulation, and this gene likely functions as a key regulator of the lignin biosynthesis pathway. Meanwhile, the induction of peroxidase genes (*Zm00001d030002* and *Zm00001d010924*) at 15 dpi was accompanied by a decrease in the intermediate guaiacylglycerol-β-guaiacyl ether glucoside, confirming the promotion of G-type lignin polymerization. The sustained upregulation of *ZmCOMT* (*Zm00001d049541*) in 92C led to the depletion of sinapic acid and the accumulation of sinapaldehyde, which drives the synthesis of S-type lignin and reduces disease resistance. Therefore, *ZmPAL* (*Zm00001d051164*), *ZmHCT* (*Zm00001d024317*), peroxidase genes (*Zm00001d030002* and *Zm00001d010924*), and *ZmCOMT* (*Zm00001d049541*) were identified as key regulatory genes mediating lignin biosynthesis in maize during FER defense, representing promising targets for maize breeding aimed at improving FER resistance.

Beyond genetic resistance, complementary biocontrol strategies have emerged as promising approaches for managing FER. A previous report indicated that combined application of the biocontrol bacterium *Ochrobactrum ciceri* with zinc can effectively inhibit *F. verticillioides* growth and fumonisin production while also improving maize seed germination, seedling growth, and disease resistance [37]. These findings complement the observations that robust lignin deposition in the resistant line ZL30 contributes to the formation of a physical barrier against fungal invasion. Together, these complementary lines of evidence suggest that integrating host genetic resistance with pathogen-targeted biocontrol strategies represents a promising approach for achieving a more effective and durable solution for controlling FER in maize.

Jasmonic acid (JA) is a well-established defense hormone that induces phenylpropanoid metabolism and promotes lignin deposition [38]. The distinct expression patterns of JA signaling components and transcription factors between ZL30 and 92C reveal the upstream regulatory network mediating lignin-mediated resistance. In the resistant line ZL30, the early activation of the JA biosynthesis gene *LOX11* (*Zm00001d015852*), together with the sustained upregulation of a JA-responsive gene (*Zm00001d034068*), indicates that JA serves as an early trigger for defense activation. Conversely, the susceptible line 92C exhibited a compromised JA response, with multiple *LOX* genes downregulated across all time points, suggesting that failure to activate effective JA-mediated defense contributes to susceptibility. MYB transcription factors, master regulators of the phenylpropanoid pathway [39], exhibited strikingly contrasting expression patterns between the two lines. In 92C, the upregulation of *MYB44*, a regulator of secondary wall biosynthesis [40], may correlate with a shift toward S-type lignin precursors and compromised cell wall integrity. In contrast, the downregulation of *MYB* expression in ZL30 likely directs metabolic flux toward G-type lignin to reinforce cell wall integrity. Collectively, these results suggest that the coordinated activation of JA signaling and the fine-tuned expression of *MYB* transcription factors in ZL30 establish an effective upstream regulatory network that directs lignin biosynthesis toward resistance-conferring cell wall reinforcement, whereas the dysregulated *MYB* expression in 92C reflects a failed defense response.

In summary, this study integrated differential gene expression enrichment analysis and metabolic profiling of two maize lines (ZL30 and 92C) inoculated with *F. verticillioides* to unveil the key metabolic pathways and genes underlying the maize defense response to FER, providing valuable insights for breeding FER-resistant maize and guaranteeing food safety. Nevertheless, several issues remain to be further investigated. First, only two maize inbred lines were used in this study, which may limit the general applicability of the results. Future studies should include a larger number of resistant and susceptible lines to validate the conserved role of the lignin biosynthesis pathway in FER resistance. Secondly, although certain genes and key pathway associated with resistance have been identified, the regulatory network among these genes requires in-depth investigation. Thirdly, while the biochemical data support the role of lignin biosynthesis in FER resistance, structural evidence such as lignin deposition patterns and cell wall thickness were not examined in this study. Future investigations incorporating microscopic analysis will be essential to confirm the structural reinforcement conferred by lignin accumulation. Fourth, although several key genes associated with lignin biosynthesis and FER resistance were identified, functional validation through reverse genetics approaches such as CRISPR/Cas9 or overexpression was not performed in this study. Future studies employing these techniques are needed to directly confirm the causal roles of these genes in conferring resistance to FER.

## 4. Materials and Methods

### 4.1. Plant Materials

*Fusarium* ear rot resistant (R) maize inbred line ZL30 (ZL30-12) and susceptible (S) maize inbred line 92C (92C0468U) were used in this study. All materials were planted in the experimental field of the Shenbei Experimental Base, Liaoning Academy of Agricultural Sciences (123.58° E, 42.02° N), with a plot design of 3 m row length and 60 cm row spacing. All plants were self-pollinated to ensure sufficient sample for subsequent experiments.

### 4.2. Inoculation Assay and Evaluation of Disease Resistance

*F. verticillioides* used in the inoculation assay was isolated, purified, identified, and preserved by Hebei Agricultural University. For inoculum preparation, the fungus was cultured on potato dextrose agar (PDA) medium in the dark at 25 °C for approximately one week until the mycelia fully covered the plates. PDA medium was prepared according to standard protocols. Briefly, 200 g of peeled potatoes was sliced, boiled in 1 L of distilled water for 30 min, and filtered through cheesecloth. The filtrate was supplemented with 20 g dextrose and 15 g agar. The pH was adjusted to 5.6, and the medium was sterilized by autoclaving at 121 °C for 15 min. Mycelial plugs were transferred to carboxymethylcellulose sodium (CMC) medium (15 g/L CMC-Na, 0.825 g/L (NH_4_)_2_SO_4_, 1 g/L KH_2_PO_4_, 1 g/L yeast extract, and 0.5 g/L MgSO_4_·7H_2_O) and incubated at 25 °C with shaking at 180 r/min for 5 days. After removing mycelia by gauze filtration, the conidial suspension was centrifuged for spore enrichment, washed twice with sterile water, adjusted to a final concentration of 1 × 10^7^ conidia/mL [41], and then stored at 4 °C for subsequent use. The wound inoculation method was adopted: at 10 days after pollination, 2 mL of the conidial suspension was injected into the middle part of each ear using a quantitative syringe. For mock-inoculated (CK), an equal volume of sterile water was injected following the same procedure as the *F. verticillioides*-inoculated group. For each line, 10 inoculated ears were selected, and the disease area was recorded. Disease severity was evaluated based on a 1–9 scale according to the percentage of diseased area per ear: 1 (0–1%), 3 (2–10%), 5 (11–25%), 7 (26–50%), and 9 (51–100%). The percentage of diseased area per ear was quantified using the IAS-MER-2123 Maize Ear Rot Intelligent Analysis System (Beijing Anjing Ruihua Technology, Beijing, China), which automatically identifies infected regions and calculates the diseased area ratio. The average disease scale for each line was calculated using the following formula:

$${\mathrm{ERS}}_{A}=\;(\sum_{i}^{n}ERS)/n$$

where ERS_A_ is the average ear rot scale, and the value of i ranges from 1 to n. The resistance level was then determined based on the average disease scale: high resistance (≤1.5), resistance (1.6–3.5), moderate resistance (3.6–5.5), susceptibility (5.6–7.5), and high susceptibility (7.6–9.0). The susceptible inbred line B73 was used as a control, and the inoculation batch was considered valid only when the average disease scale of B73 was ≥5.6 [41]. Kernels surrounding the inoculation site were collected from each ear as one biological replicate. Three biological replicates were collected per treatment for subsequent analysis.

### 4.3. Determination of Fumonisin Content

Fumonisin content in kernels of ZL30 and 92C at different post-inoculation time points was determined by high-performance liquid chromatography–tandem mass spectrometry (LC-MS/MS) using an Agilent Ultivo Triple Quad LC/MS 1290-6465 system (Agilent Technologies, Santa Clara, CA, USA). Kernel samples were ground into powder and passed through a 40-mesh sieve. A 2.5 g aliquot of the powder was added to 50 mL of methanol–water solution (3:1, *v*/*v*) and homogenized thoroughly. After standing for 10 min, the supernatant was collected and centrifuged at 1800 rpm for 5 min. The supernatant was then filtered, and the pH of the filtrate was adjusted to 5.8–6.5. The filtrate was purified using Bond Elut SAX solid-phase extraction columns (Agilent Technologies, Santa Clara, CA, USA) (500 mg, 3 mL). The column was preconditioned sequentially with 5 mL of methanol and 5 mL of methanol–water (3:1, *v*/*v*). An aliquot of 2 mL of the filtrate was then loaded at a flow rate below 2 mL/min. The column was washed successively with 5 mL of methanol–water (3:1, *v*/*v*) and 3 mL of methanol. Fumonisins were eluted with 9 mL of 1% formic acid in methanol at a flow rate below 1 mL/min. The eluate was evaporated to dryness under a gentle stream of nitrogen, and the residue was reconstituted in 0.5 mL of acetonitrile–water (1:1, *v*/*v*), vortexed for 30 s, and filtered through a 0.22 µm organic membrane prior to LC-MS/MS analysis. The fumonisin contents were quantified by an Agilent Ultivo Triple Quad LC/MS 1290-6465 system (Agilent Technologies, Santa Clara, CA, USA) under optimized chromatographic conditions [42].

### 4.4. Transcriptome Sequencing and Data Processing

Total RNA was extracted from maize kernels using Trizol^®^ reagent (Invitrogen, Waltham, MA, USA), with three independent biological replicates per sample. The quantity and quality of the extracted RNA were evaluated using a NanoDrop 1000 spectrophotometer (NanoDrop Technologies, Wilmington, DE, USA). cDNA library construction and high-throughput sequencing were performed by Wuhan Metware Biotechnology (Wuhan, China). To obtain high-quality clean reads, raw FASTQ reads were processed with fastp [43] to remove low-quality sequences and adapter contaminants. The filtered clean reads were mapped to the maize reference genome (Maize GDB, v 4.0) using HISAT2 [44]. Differentially expressed genes (DEGs) were identified using DESeq2 with the screening criteria of padj < 0.05 and |log_2_FC| > 1. Heatmap analysis of DEGs was performed using R (v3.2.0) following Z-score normalization of the expression data to visualize the gene expression patterns across different groups and samples. Gene Ontology (GO) enrichment analysis of the DEGs was conducted using the DAVID tool (v 6.8) [45] to predict their potential biological functions.

### 4.5. Metabolomic Analysis

Kernel samples (three biological replicates per group) were lyophilized using a vacuum freeze-dryer (Scientz-100F, Ningbo Scientz Biotechnology, Ningbo, China). The lyophilized samples were ground into a fine powder using a mixer mill (MM 400, Retsch GmbH, Haan, Germany) at 30 Hz for 1.5 min. Subsequently, 50 mg of the lyophilized powder was extracted with 1200 µL of pre-chilled 70% (*v*/*v*) methanol solution. After centrifugation at 12,000 rpm for 3 min, the supernatant was filtered through a 0.22 µm organic filter membrane (SCAA-104, ANPEL Laboratory Technologies, Shanghai, China) prior to liquid chromatography–mass spectrometry (LC-MS) analysis. All metabolic profiling analyses were conducted by Wuhan Metware Biotechnology (Wuhan, China). Differentially accumulated metabolites were identified using the criteria of variable importance in projection (VIP) > 1 and FC ≥ 2 or FC ≤ 0.5. The reproducibility of metabolomic data was validated by PCA analysis, confirming the reliability of three biological replicates.

### 4.6. Measurement of Lignin Content

Lignin content was determined using a commercial lignin assay kit following the manufacturer’s protocols. Briefly, kernel samples were oven-dried at 80 °C to a constant weight, ground into a fine powder, and passed through a standard sieve. A 2 mg aliquot of the dried powder was placed into a 10 mL glass test tube. Subsequently, 500 µL of Reagent 1 and 20 µL of perchloric acid were added to both blank and sample tubes. The tubes were sealed with parafilm, incubated in an 80 °C water bath for 40 min with gentle shaking at 10 min intervals, and then allowed to cool naturally to room temperature. After adding 500 µL of Reagent 2 and mixing thoroughly, 20 µL of the supernatant was transferred to a new centrifuge tube, and 980 µL of Reagent 3 was added and mixed well. Finally, 200 µL of the mixture was transferred to a micro quartz cuvette or 96-well UV plate, and the absorbance at 280 nm was measured using a UV–visible spectrophotometer (Shimadzu Corporation, Kyoto, Japan) or microplate reader (Bio-Rad Laboratories, Hercules, CA, USA).

### 4.7. RT-qPCR

Kernel samples were harvested 5, 10, and 15 days post-inoculation (dpi) from *F. verticillioides*-inoculated (inoculated) and water-inoculated (CK) plants of both ZL30 and 92C maize inbred lines. Total RNA was extracted using the Fast King RT Kit (TIANGEN, Beijing, China), and first-strand cDNA was subsequently synthesized from the total RNA using the Super Real PreMix Plus kit (TIANGEN, Beijing, China). Six DEGs were selected for validation by retrotranscribed quantitative PCR (RT-qPCR) analysis. The relative expression levels of target genes were calculated using the 2^−ΔΔCt^ method [46], with *ZmACTIN* as the internal reference gene. All primers used in this study are listed in Table S16. RT-qPCR data were analyzed using Student’s *t*-test for statistical significance (*, *p* < 0.05; **, *p* < 0.01).

### 4.8. Statistical Analysis

All experiments were performed with three independent biological replicates. Data are presented as the mean ± standard deviation (SD). Statistical analyses were performed using R software (version 4.0). Student’s *t*-test was used to assess the significant difference between the two groups. A *p*-value < 0.05 was considered statistically significant.

## 5. Conclusions

*Fusarium* ear rot (FER) is a destructive disease threatening maize production and food safety worldwide. In this study, integrated transcriptomic and metabolomic analyses revealed that the resistant maize line ZL30-12 (ZL30) inhibits fungal infection and fumonisin accumulation by stably activating the phenylpropanoid–lignin biosynthesis pathway, while the susceptible line 92C0468U (92) displays dysregulated gene expression and disturbed metabolic flux in this pathway, leading to reduced lignin accumulation and compromised resistance. Key genes, including *ZmPAL*, *ZmHCT*, *ZmCOMT*, and *peroxidase* genes, were identified as critical regulators. These findings demonstrate that coordinated phenylpropanoid–lignin biosynthesis contributes importantly to FER resistance, providing valuable gene targets and theoretical support for maize resistance breeding.

## Data Availability

The data presented in this study are openly available in GSA at https://ngdc.cncb.ac.cn/gsa/browse/CRA039728, reference number CRA039728, accessed on 8 April 2026.

## Supplementary Materials

The following supporting information can be downloaded at: https://www.mdpi.com/article/10.3390/plants15081148/s1, Figure S1: Disease severity and fumonisin accumulation in ZL30 and 92C following *F. verticillioides* inoculation. (**A**) Percentage of infected area of ear in ZL30 and 92C at 5, 10 and 15 days post inoculation (dpi); (**B**–**D**) Conent of Fumonisin B1 (**B**), Fumonisin B2 (C), and Fumonisin B3 (**D**) in ZL30 and 92C at 5, 10, and 15 dpi. Data are presented as mean ± SD. Asterisks indicate significant differences between ZL30 and 92C at each time point (**, *p *< 0.01); Figure S2: GO enrichment analysis of DEGs in ZL30 (**A**) and 92C (**B**) in response to *F. verticillioides* infection at 5 dpi; Figure S3: Principal component analysis (PCA) of metabolomic profiles; Table S1: Differentially expressed genes in ZL30 at 5 dpi upon *F. verticillioides* inoculation; Table S2: Differentially expressed genes in ZL30 at 10 dpi upon *F. verticillioides* inoculation; Table S3: Differentially expressed genes in ZL30 at 15 dpi upon *F. verticillioides* inoculation; Table S4: Differentially expressed genes in 92C at 5 dpi upon *F. verticillioides inoculation*; Table S5: Differentially expressed genes in 92C at 10 dpi upon *F. verticillioides* inoculation; Table S6: Differentially expressed genes in 92C at 15 dpi upon *F. verticillioides* inoculation; Table S7: Differentially accumulated metabolites in ZL30 at 10 dpi upon *F. verticillioides* inoculation; Table S8: Differentially accumulated metabolites in ZL30 at 5 dpi upon *F. verticillioides* inoculation; Table S9: Differentially accumulated metabolites in ZL30 at 15dpi upon *F. verticillioides* inoculation; Table S10: Differentially accumulated metabolites in 92C at 10 dpi upon *F. verticillioides* inoculation; Table S11: Differentially accumulated metabolites in 92C at 5 dpi upon *F. verticillioides* inoculation; Table S12: Differentially accumulated metabolites in 92C at 15 dpi upon *F. verticillioides* inoculation; Table S13: DEGs unique to resistant line ZL30 and susceptible line 92C; Table S14: DAMs unique to resistant line ZL30 and susceptible line 92C; Table S15: Correlation analysis of gene expression and metabolite accumulation in the lignin biosynthesis pathway; Table S16: List of primers used in this study.

## Abbreviations

The following abbreviations are used in this manuscript:

FER	*Fusarium* ear rot
QTL	Quantitative trait locus
FB1	Fumonisin B1
FB2	Fumonisin B2
FB3	Fumonisin B3
DEGs	Differentially expressed genes
DAMs	Differentially accumulated metabolites
