# Supplementary material for: Integrated Transcriptomic and Metabolic Analyses Reveal Key Defense Pathways Against Fusarium Infection in Maize Kernels
Source: Plants (Basel). 2026 Apr 9;15(8):1148. doi: 10.3390/plants15081148 (PMC13118878; doi:10.3390/plants15081148)
Supplement: Supplementary file 1 [file plants-15-01148-s001.zip › Figure S1.pdf]

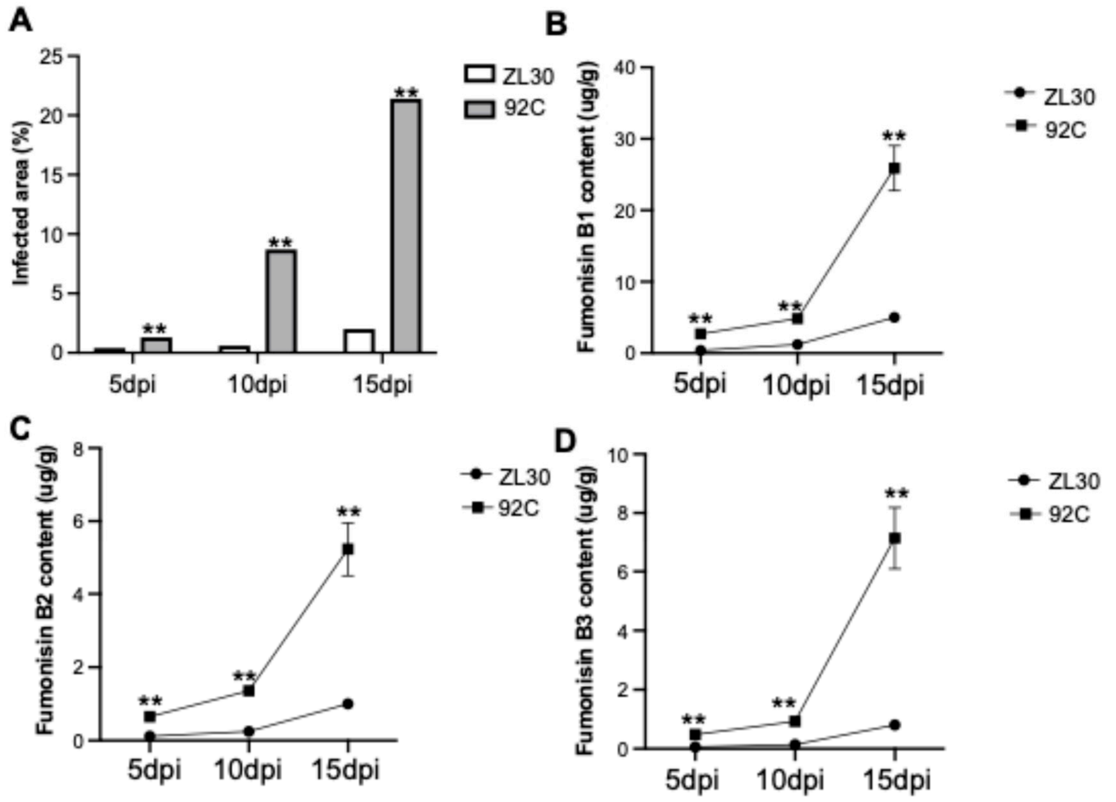

Figure S1. Disease severity and fumonisin accumulation in ZL30 and 92C post *F. verticillioide*s inoculation. (A) Percentage of infected area of ear in ZL30 and 92C at 5, 10 and 15 days post inoculation (dpi); (B-D) Content of Fumonisin B1 (B), Fumonisin B2 (C), and Fumonisin B3 (D) in ZL30 and 92C at 5, 10, and 15 dpi. Data are presented as mean ± SD. Asterisks indicate significant differences between ZL30 and 92C at each time point (**, $P < 0.01$).
