# Supplementary material for: Integrated Transcriptomic and Metabolic Analyses Reveal Key Defense Pathways Against Fusarium Infection in Maize Kernels
Source: Plants (Basel). 2026 Apr 9;15(8):1148. doi: 10.3390/plants15081148 (PMC13118878; doi:10.3390/plants15081148)
Supplement: Supplementary file 1 [file plants-15-01148-s001.zip › Figure S2.pdf]

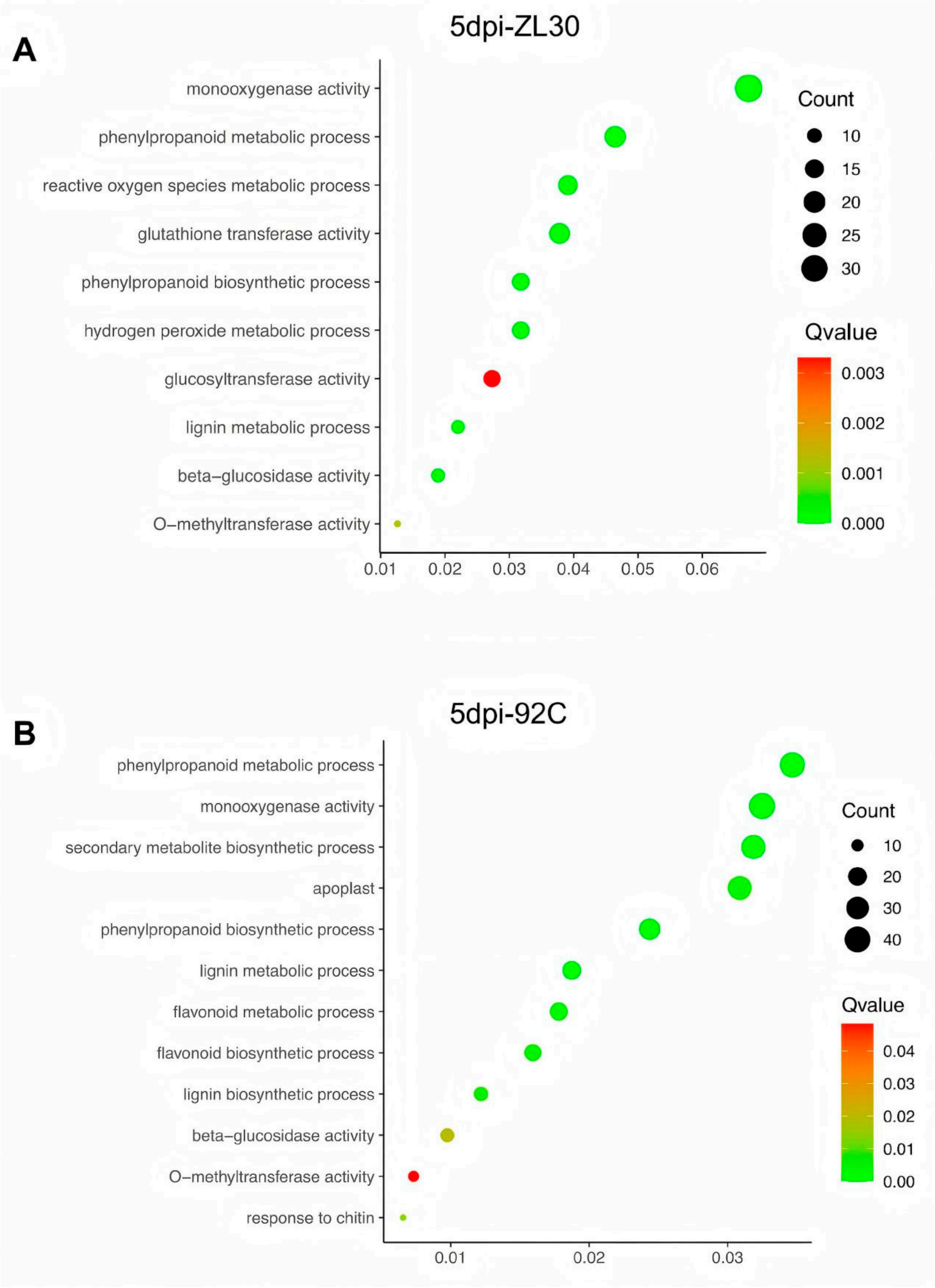

Figure S2. GO enrichment analysis of DEGs in ZL30 (A) and 92C (B) in response to *F. verticillioides* inoculation at 5 dpi.
