# Supplementary figures and images for: Integrated Transcriptomic and Metabolic Analyses Reveal Key Defense Pathways Against Fusarium Infection in Maize Kernels
Source: Plants (Basel). 2026 Apr 9;15(8):1148. doi: 10.3390/plants15081148 (PMC13118878; doi:10.3390/plants15081148)

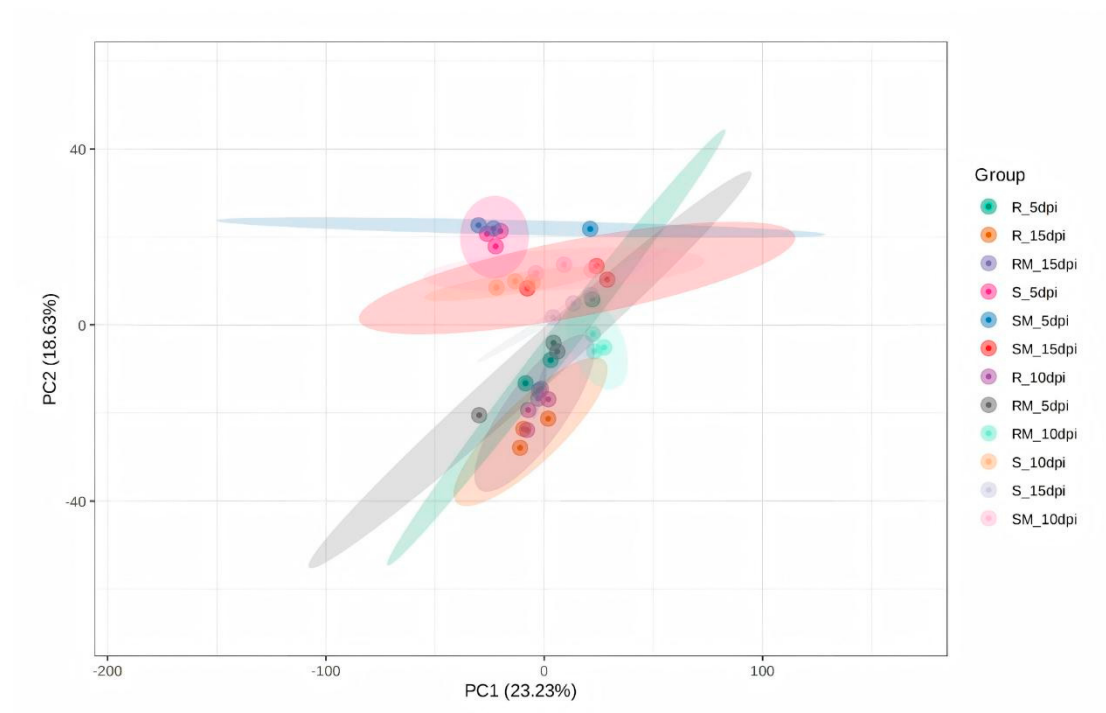

**Figure S3.** Principal component analysis (PCA) of metabolomic profiles.

Supplement: Supplementary file 1 [file plants-15-01148-s001.zip › Figure S3.pdf]
